# The Interplay of the Gut Microbiome, Bile Acids, and Volatile Organic Compounds

**DOI:** 10.1155/2015/398585

**Published:** 2015-03-03

**Authors:** Nidhi M. Sagar, Ian A. Cree, James A. Covington, Ramesh P. Arasaradnam

**Affiliations:** ^1^Warwick Medical School, University of Warwick, Coventry CV4 7AL, UK; ^2^Clinical Sciences Research Institute, University of Warwick, Coventry CV4 7AL, UK; ^3^School of Engineering, University of Warwick, Coventry CV4 7AL, UK

## Abstract

*Background*. There has been an increasing interest in the use of volatile organic compounds (VOCs) as potential surrogate markers of gut dysbiosis in gastrointestinal disease. Gut dysbiosis occurs when pathological imbalances in gut bacterial colonies precipitate disease and has been linked to the dysmetabolism of bile acids (BA) in the gut. BA metabolites as a result of microbial transformations act as signaling molecules and have demonstrated regulation of intestinal homeostasis through the TGR5 and FXR receptors by inhibiting inflammation, preventing pathogen invasion, and maintaining cell integrity. The presence of VOC footprints is the resultant effect to gut microbiome substrate fermentation. *Aim*. To review the role of the gut microbiome and bile acid signaling in intestinal homeostasis and the resultant use of VOCs as potential noninvasive surrogate biomarkers in gut dysbiosis. *Methods*. A systematic search on PubMed and Medline databases was performed to identify articles relevant to gut dysbiosis, BA metabolism, and VOCs. *Conclusions*. The host and presence of the gut microbiome appear to regulate the BA pool size. A dysbiotic gut microbiome results in disrupted intestinal homeostasis, which may be reflected by VOCs, differentiating those who are healthy and those with disease.

## 1. Introduction

The human intestinal microbiota has been the subject of extensive interest in recent years however this complex ecosystem remains incompletely characterized and ongoing research aims to develop a clearer understanding of its fundamental role in health and disease.

The gastrointestinal tract harbors a diverse community of approximately 10^4^ microorganisms comprising 500 to 1000 distinct bacterial species and is the most heavily colonized organ in the human body [1]. Anaerobic bacteria constitute the majority of the gut microbiota and are predominantly represented by* Bacteroidetes* and the* Firmicutes*, with* Proteobacteria*,* Verrucomicrobia*,* Actinobacteria*,* Fusobacteria*, and Cyanobacteria being present in minimal proportions [2]. The composition of the microbiota is unique to each individual and it has been demonstrated that the faecal microbiome of identical twins shares less than 50% of species phylotypes [3]. In the elderly population, there are age-related physiological changes in the gut microbiota, which may result in a microbial imbalance due to chronic low-grade inflammation [4, 5]. High throughput sequencing analysis has demonstrated a differing composition of gut microbiota of older people (above 65 years) compared to younger people with a predominance of the phylum* Bacteroidetes* [6].* Bacteroidetes* have been identified primarily in residents in long-stay care environments and these individuals demonstrated considerably less diverse microbiota with loss of the community-associated flora, which was associated with frailty [7]. This supports the implication that the gastrointestinal microbiota is extremely significant in the health and development of disease in the older population.

## 2. Gut Microbiome and Health

The gut microbiota contains at least 100 times as many genes as the human genome, most of which confer physiological functions. These recognized roles include metabolic functions such as vitamin synthesis, regulating the uptake and deposition of dietary lipids, absorbing indigestible carbohydrates, and modulating the intestinal epithelium's absorptive capacity for optimum nutrient metabolism. Protective functions incorporate the maintenance of intestinal barrier integrity and barricading against invading pathogens by competitive exclusion through production of antimicrobial peptides, engagement of attachment sites, and consumption of nutrient supplies. Immunomodulation functions include tolerance to dietary and microbial antigens. This is mediated by the induction of regulatory T cells as well as inhibiting overgrowth of the gut microbiota and translocation to systemic sites through activating intestinal dendritic cells (DCs), which selectively induces the production of IgA from plasma cells. Despite infiltration of the lamina propria with activated immune cells and only a single epithelial layer allowing for separation from the gut microbiota, healthy individuals do not demonstrate pathological features. Therefore, regulatory mechanisms exist not only to ensure intestinal immune homeostasis in a healthy gut but also to stimulate a protective immune response in the presence of pathogen invasion. Small numbers of live commensal organisms penetrate Peyer's patches and the bacterial antigens are taken up by the DCs resulting in mucosal immune responses and induction of IgA B cells. These B cells occupy the lamina propria by recirculating through the lymph and bloodstream to secrete protective IgA. This protects against mucosal penetration of bacteria as the DCs loaded with bacteria are confined to the mucosal immune compartment by the mesenteric lymph nodes, ensuring local induction of immune responses to the bacteria while the systemic immune system remains relatively ignorant of these organisms [8–11].

**Figure 1:**
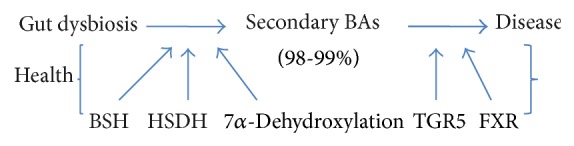
Proposed schema of interplay between gut dysbiosis, modified BA pool, and disease. In health, secondary BAs are modified by microbial BSH and HSDH enzymes through deconjugation, oxidation, and epimerization as well as dehydroxylation via 7*α*-dehydroxylation activity. The BA metabolites as a result of microbial transformations act as signaling molecules via the TGR5 and FXR receptors to regulate intestinal homeostasis. In disease, it is unclear how gut dysbiosis causes a modified BA pool, which then results in disease, which is possibly secondary to impaired BA signaling. (BSH: bile salt hydrolases; HSDH: hydroxysteroid dehydrogenases; TGR5: G protein coupled BA receptor; FXR: farnesoid x receptor).

Dysbiosis occurs when pathological imbalances in gut bacterial colonies precipitate disease and has been linked to the dysmetabolism of bile acids (BA) in the gut (Figure 1).

## 3. Gut Microbiome and Bile Acid Metabolism

BAs are saturated, hydroxylated C24 cyclopentanophenanthrene sterols and are the main facilitators of lipid absorption in the gastrointestinal tract [1]. Cholic acid (CA) and chenodeoxycholic acid (CDCA) are the two primary BAs synthesized in the liver. BAs are further metabolized by conjugation (*N*-acyl amidation) in the liver to glycine or taurine. They are then actively secreted across the canalicular membrane and carried in bile to the gallbladder where they are stored [12]. Secretin and cholecystokinin (CCK) are secreted by chyme from an ingested meal. Biliary duct cells are stimulated by secretin to secrete bicarbonate and water to increase the volume of bile. CCK stimulates gallbladder contraction causing bile to flow into the duodenum [13]. The primary BAs then activate the FXR in the liver which stimulates expression of small heterodimer partner (SHP) to inhibit the action of the homolog-1 liver receptor which controls the upregulation of the rate-limiting BA synthesis enzyme CYP7A1 [14, 15]. BA synthesis is also inhibited through intestinal FXR activity, which stimulates the expression of fibroblast growth factor 19 (FGF19). FGF19 binds hepatic FGF4 and activates c-Jun N-terminal kinase 1/2 (JNK1/2) and extracellular signal-regulated kinase 1/2 (ERK1/2) to impede BA synthesis [16]. Bile acids are actively reabsorbed from the ileum and circulated back to the liver via the hepatic portal vein. This process is known as the enterohepatic circulation and ensures recycling of the majority of synthesized BAs to maintain a functional BA pool.

Only 1-2% of BAs escape the enterohepatic circulation and undergo microbial biotransformation in the large bowel to form the secondary BAs, deoxycholic acid (DCA), and lithocholic acid (LCA) [12]. Transformations include deconjugation (removal of the amino acid side chain), epimerization (of 3-, 7-, and 12-hydroxy groups), oxidation (removal of H_2_), dehydroxylation (replacement of a hydroxyl group with a hydrogen), and hydroxylation (replacement of a hydrogen with a hydroxyl group) [13]. This microbial transformation is a key step and is discussed in detail below.

### 3.1. Deconjugation

BSHs are enzymes that catalyse the hydrolysis of the C24 N-acyl amide bond of conjugated BAs. BSH enzymes have been purified from* Bacteroides fragilis*,* Bacteroides vulgatus*,* Clostridium perfringens*,* Listeria monocytogenes*, and several species of* Lactobacillus* and* Bifidobacterium* [1, 17]. There are currently three main hypotheses with regard to the ecological importance of microbial BSH activity. Firstly, the liberated amino acids from deconjugation may potentially be used as carbon, nitrogen, and energy sources. Glycine may be metabolized to ammonia and carbon dioxide and taurine to ammonia, carbon dioxide, and sulphate, which could then be integrated into bacterial metabolites [13]. Secondly, the tensile strength of the membranes may be increased or a change in membrane fluidity may be affecting sensitivity to *α*-defensins and other host defense molecules via BSHs facilitating incorporation of cholesterol or bile into bacterial membranes [18–20]. Thirdly, BSHs may play a role in bile tolerance and serve as a detoxification method, allowing for microbial survival in the gastrointestinal tract in the presence of bile salts [13].

### 3.2. Oxidation and Epimerization

Oxidation and epimerization of the 3-, 7-, and 12-hydroxy groups of BAs in the gastrointestinal tract are catalyzed by HSDHs expressed by intestinal bacteria. Epimerization of BA hydroxyl groups generates a stable oxo-bile acid intermediate and requires the actions of two stereochemically distinct HSDHs and can be performed by a single species containing both *α*- and *β*-HSDHs or by two species, one possessing an *α*-HSDH and the other a *β*-HSDH. 3-*α* and 3-*β* HSDHs have been identified in several bacteria belonging to the* Firmicutes* phylum whereas bacteria capable of intraspecies 3-hydroxy epimerization include* Peptostreptococcus productus*,* C. perfringens*, and* Eggerthella lenta*. 7*α*-HSDHs have been detected among members of the* Clostridium*,* Eubacterium*,* Bacteroides*, or* Escherichia* genera. Intraspecies 7-hydroxy epimerization has been observed in species of the* Clostridium*,* Eubacterium*, and* Ruminococcus* genera [13].

### 3.3. Dehydroxylation

The 7*α*-dehydroxylation of primary BAs results in the formation of the secondary BAs, which predominate in human faeces. Therefore, this is the most quantitatively important microbial bile salt transformation. Species of the* Firmicutes* phylum (*Clostridium* and* Eubacterium*) possess 7*α*-dehydroxylation activity [13]. 7*α*/*β*-dehydroxylation is restricted to free Bas; therefore removal of glycine/taurine BA conjugates via BSH enzymes is a precondition for 7*α*/*β*-dehydroxylation [21–23].

## 4. Bile Acid Signalling

BA metabolites as a result of microbial transformations act as signaling molecules and have demonstrated regulation of intestinal homeostasis through the TGR5 and FXR by inhibiting inflammation, preventing pathogen invasion, and maintaining cell integrity. TGR5 is principally activated by secondary BAs, including DCA and LCA. This receptor minimizes production of proinflammatory cytokines (IL-1*α*, IL-2*β*, IL-6, and TNF*α*) stimulated by lipopolysaccharides in macrophages and Kupffer cells through inhibition of NF-kB [24]. Activation of the BA receptor FXR protects against bacterial overgrowth and translocation in the distal small intestine and resultant disruption to the gut epithelial barrier through the regulation of several genes, including Ang1, Inos, and IL18, which have recognized antimicrobial actions [25]. The degree of activation of BA receptors is influenced primarily by the gut microbiota and therefore dysbiosis may result in abnormal BA modification resulting in the development of gastrointestinal disease.

Uncontrolled levels of BAs may exert detrimental health effects. Abnormally increased concentrations of hydrophobic secondary BAs are cytotoxic, causing DNA damage and cell death through the likely mechanism of induction of oxidative stress and production of reactive oxygen species [26, 27]. BAs are important regulators of gut homeostasis with antimicrobial and amphipathic properties. DCA at a concentration of 0.5 mM can successfully prevent bacterial growth in cell culture demonstrating regulation of gut microbial composition through environmental stress [28]. 10–100 trillion microbes inhabit the human gastrointestinal tract providing effective metabolic activity to process undigestible dietary sources with resultant disruptions to the gut microbiota leading to impaired metabolism and nutrient acquisition as well as potential for pathogen invasion.

### 4.1. Effect of Diet

Diet has been demonstrated to have a considerable influence on microbial composition, function, and effects. Mice fed a high fat diet were found to have impaired intestinal mucosal barrier integrity secondary to modification of the BA profile with an increase in the concentration of DCA and decrease in the proportion of a potentially cytoprotective tertiary BA, ursodeoxycholic acid (UDCA). The decrease in UCDA was associated with disruption of the intestinal barrier most likely due to the increased ability of cytotoxic BAs like DCA to induce barrier dysfunction. DCA has been recognised to disrupt lipid bilayers while the hydrophilic BA UCDA stabilizes them and protects mitochondria against DCA-induced reactive oxygen species production [29]. Another mouse study found that consumption of a high saturated fat diet stimulated the expansion of the sulphite-reducing microorganism,* Bilophila wadsworthia*. This was related to a proinflammatory T helper type 1 immune response with an increased incidence of colitis in genetically susceptible mice that lacked IL-10 [30].

### 4.2. Lipid and Cholesterol Metabolism

Through activation of the FXR and TGR5 receptors, BAs have shown a significant role in lipid haemostasis. Treatment of cholesterol gallstones with CDCA has been demonstrated to reduce plasma triglyceride levels and hepatic VLDL production [31]. Evidence for the mechanism behind this has been reported in a recent mouse study where BAs, by activating FXR, induce the expression of SHP, an atypical nuclear receptor [32]. FXR represses the hepatic expression of the genes phosphoenolpyruvate carboxykinase and glucose-6-phosphatase which are involved in gluconeogenesis [33]. SHP inhibits the activity of the liver X receptor (LXR) and other transcription factors, which are essential for the transcription of CYP7A1 (the rate-limiting enzyme in BA biosynthesis) and stimulate SREBP-1c expression [32]. SREBPs have a role in controlling genes that regulate biosynthesis of cholesterol and its receptor-mediated uptake from LDL. In addition, SREBPs also govern the expression of genes such as fatty acid synthase, acetyl-CoA carboxylase, and glycerol-3-phosphate acyltransferase which are involved in lipogenesis [34].

The gut microbiota, bile acids, and health status are closely integrated and influence each other making it difficult to ascertain whether gut dysbiosis and modified BA pools are a cause or consequence of disease.

## 5. Irritable Bowel Syndrome (IBS) and the Gut Microbiome

The pathophysiology of IBS remains incompletely understood but may involve altered gut microbiome. The existence of abnormal colonic fermentation (increased hydrogen colonic gas production seen in IBS patients compared to controls), improvement with antibiotic therapy in 48% of patients with both small intestinal bacterial overgrowth and IBS, and a high incidence of IBS after gastrointestinal infections imply a role for intestinal microbiota in IBS as acute enteritis is associated with an increase in mucosal cytotoxic T lymphocytes and an increase in enteroendocrine hypersensitivity which will impact the gut microbiota environment [35–38]. An augmented cellular immune response with production of the proinflammatory cytokines including TNF-alpha, IL-1, and IL-6 in patients with diarrhea predominant IBS also supports the role of gut microbiota in the aetiology of IBS [39]. In addition, significantly elevated levels of human beta-defensin-2 (expression induced by proinflammatory cytokines and probiotic microorganisms) were characterized in patients with active IBS compared to healthy controls, signifying an activation of the mucosal innate defense system towards a proinflammatory response [40]. Evidence of the presence of an immune association between the gut microbiota and host in IBS has been shown by the increased expression of Toll-like receptors 4 and 5, a family of pathogen-recognition receptors of the innate immune system, in IBS patients [41]. Further support of potential association of dysbiosis in IBS is suggested by treatment with probiotic therapy using* Lactobacillus plantarum* 299V, or the VSL3 capsule (mixture of* lactobacilli* and* bifidobacteria*) [42–44]. These treatments have demonstrated an improvement in IBS symptoms (though not sustained), in particular abdominal pain and bloating which emphasizes the known ability of probiotics in balancing intestinal microbiota.

The composition of the gut microbiota has been found to reflect symptom severity in IBS with the presence of the* Ruminococcus torques* phylotype being associated with an increase in severity of bowel symptoms.* R torques* is a recognized mucin degrader; therefore this fact may account for the reported increase in mucin in the context of IBS [45]. Using PCR and culture based techniques, the gut flora of IBS patients have been characterized to reveal reduced numbers of* bifidobacteria* and* lactobacilli* and an increased aerobe to anaerobe ratio [46–48]. Mucosal bacteria, including* Eubacterium rectale* and* Bacteroides* (*Prevotella*,* fragilis*, and* distasonis*), have also been shown to be more abundant in IBS patients compared to controls [49].

### 5.1. Gut Microbiome, BA Dysmetabolism, and IBS

Modifications in faecal BA composition in diarrhoea predominant IBS (IBS-D) patients have been demonstrated with a significant increase in primary BA and a parallel decrease in secondary BA compared to healthy controls. This finding correlated with a higher stool frequency and a lower stool consistency as measured by the Bristol stool chart. This may reflect the influence of dysbiosis found in this study with IBS-D patients exhibiting a decrease in* Bifidobacterium*, an increase in* E. coli*, and lower counts of* leptum*. The* leptum* group contains many bacteria (in particular* Ruminococcus* and* Clostridia*) which are involved in BA transformation; therefore lower numbers of these bacteria may account for the reduced transformation activity of the microbiota, resulting in increased primary BAs and reduced secondary BAs [50, 51]. This would suggest that the altered gut microbiome is the primary driver for BA dysregulation in those with IBS. Results from this study are supported by another randomized controlled study where sodium chenodeoxycholate (CDCA), a primary BA, was given to healthy subjects and was found to significantly accelerate colonic transit, increase stool frequency, and decrease stool consistency, suggesting that excess CDCA stimulates diarrhoea in IBS patients [52].

## 6. Volatile Organic Compounds (VOCs)

VOCs are a diverse group of carbon-based chemicals that are volatile at ambient temperature [53]. They exist in the gaseous phase and are present in faeces, urine, exhaled air, and sweat. Fermentation of nonstarch polysaccharides by gut microbiota produces an odorous gas composed of various VOCs. Bacteroides in particular have been shown to produce ethanoic, propionic, butanoic, pentanoic, and hexanoic acids [54]. Colonic fermentation is controlled by the colonocyte, colonic bacteria, and diet. It is thought that VOCs are shared by individuals in health with specific changes occurring in disease. The resultant VOC profile, which reflects microbial metabolic activity, is a specific biomarker of colonic as well as metabolic disease. Currently, there is limited evidence on the use of VOCs as potential noninvasive biomarkers in gastrointestinal disease; however, the results from the few studies available appear promising. VOCs have been found to separate out those patients with inflammatory bowel disease (IBD) compared to controls and also distinguish between active IBD and those in remission [55]. Additionally, VOC profiles have been found to differ in patients with bile acid diarrhoea (BAD), ulcerative colitis, and controls with specific chemical compounds being identified in BAD [56]. The presence of VOC footprints is the resultant effect to gut microbiome substrate fermentation and this may hold potential as a surrogate marker for intestinal dysbiosis as evidenced by the recent studies described above.

## 7. Concluding Remarks

The host and presence of the gut microbiome appear to regulate the BA pool size. Through bile salt hydrolysis and bile acid 7*α*-dehydroxylation, microbes in the gut are proficient in producing secondary BAs that bind to and activate a number of host nuclear receptors, affecting host physiology. A dynamic equilibrium is evident between diet, the gut microbiome, and the size and composition of the BA pool. A dysbiotic gut microbiome arising from diet, antibiotic therapy, or disease results in disrupted intestinal homeostasis. VOCs are potential noninvasive biomarkers, which may reflect gut dysbiosis, differentiating those in health and with disease. Unravelling the complex interactions between the host, Bas, and the gut microbiome and developing further studies to observe gut dysbiosis and its influence on VOC production will enable firstly the development of new approaches to diagnose certain gastrointestinal conditions and secondly modulating the host-microbiome-bile acid axis for therapeutic interventions.

## References

[1] Gérard P. (2014). Metabolism of cholesterol and bile acids by the gut microbiota. *Pathogens*.

[2] Eckburg P. B., Bik E. M., Bernstein C. N. (2005). Diversity of the human intestinal microbial flora. *Science*.

[3] Turnbaugh P. J., Quince C., Faith J. J. (2010). Organismal, genetic, and transcriptional variation in the deeply sequenced gut microbiomes of identical twins. *Proceedings of the National Academy of Sciences of the United States of America*.

[4] Franceschi C. (2007). Inflammaging as a major characteristic of old people: can it be prevented or cured?. *Nutrition Reviews*.

[5] Guigoz Y., Doré J., Schiffrin E. J. (2008). The inflammatory status of old age can be nurtured from the intestinal environment. *Current Opinion in Clinical Nutrition and Metabolic Care*.

[6] Claesson M., Cusack S., O'Sullivan O. (2011). Composition, variability, and temporal stability of the intestinal microbiota of the elderly. *Proceedings of the National Academy of Sciences of the United States of America*.

[7] Claesson M. J., Jeffery I. B., Conde S. (2012). Gut microbiota composition correlates with diet and health in the elderly. *Nature*.

[8] Aziz Q., Doré J., Emmanuel A., Guarner F., Quigley E. M. M. (2013). Gut microbiota and gastrointestinal health: current concepts and future directions. *Neurogastroenterology and Motility*.

[9] Macpherson A. J., Uhr T. (2004). Induction of protective IgA by intestinal dendritic cells carrying commensal bacteria. *Science*.

[10] Tsuji M., Suzuki K., Kinoshita K., Fagarasan S. (2008). Dynamic interactions between bacteria and immune cells leading to intestinal IgA synthesis. *Seminars in Immunology*.

[11] MacDonald T. T., Monteleone I., Fantini M. C., Monteleone G. (2011). Regulation of homeostasis and inflammation in the intestine. *Gastroenterology*.

[12] Vlahcevic Z. R., Heuman D. M., Hylemon P. B., Zakim D., Boyer T. (1996). Physiology and pathophysiology of enterohepatic circulation of bile acids. *Hepatology: A Textbook of Liver Disease*.

[13] Begley M., Gahan C. G. M., Hill C. (2005). The interaction between bacteria and bile. *FEMS Microbiology Reviews*.

[14] Goodwin B., Jones S. A., Price R. R. (2000). A regulatory cascade of the nuclear receptors FXR, SHP-1, and LRH-1 represses bile acid biosynthesis. *Molecular Cell*.

[15] Wei J., Qiu D. K., Ma X. (2009). Bile acids and insulin resistance: implications for treating nonalcoholic fatty liver disease. *Journal of Digestive Diseases*.

[16] Tsuei J., Chau T., Mills D., Wan Y.-J. Y. (2014). Bile acid dysregulation, gut dysbiosis, and gastrointestinal cancer. *Experimental Biology and Medicine*.

[17] Jones B. V., Begley M., Hill C., Gahan C. G. M., Marchesi J. R. (2008). Functional and comparative metagenomic analysis of bile salt hydrolase activity in the human gut microbiome. *Proceedings of the National Academy of Sciences of the United States of America*.

[18] Dambekodi P. C., Gilliland S. E. (1998). Incorporation of cholesterol into the cellular membrane of Bifidobacterium longum. *Journal of Dairy Science*.

[19] Taranto M. P., Murga M. L. F., Lorca G., de Valdez G. F. (2003). Bile salts and cholesterol induce changes in the lipid cell membrane of *Lactobacillus reuteri*. *Journal of Applied Microbiology*.

[20] Taranto M. P., Sesma F., Holgado A. P. D. R., de Valdez G. F. (1997). Bile salts hydrolase plays a key role on cholesterol removal by *Lactobacillus reuteri*. *Biotechnology Letters*.

[21] Stellwag E. J., Hylemon P. B. (1979). 7*α*-dehydroxylation of cholic acid and chenodeoxycholic acid by *Clostridium leptum*. *The Journal of Lipid Research*.

[22] Batta A. K., Salen G., Arora R., Shefer S., Batta M., Person A. (1990). Side chain conjugation prevents bacterial 7-dehydroxylation of bile acids. *The Journal of Biological Chemistry*.

[23] White B. A., Lipsky R. L., Fricke R. J., Hylemon P. B. (1980). Bile acid induction specificity of 7*α*-dehydroxylase activity in an intestinal *Eubacterium* species. *Steroids*.

[24] Keitel V., Donner M., Winandy S., Kubitz R., Häussinger D. (2008). Expression and function of the bile acid receptor TGR5 in Kupffer cells. *Biochemical and Biophysical Research Communications*.

[25] Inagaki T., Moschetta A., Lee Y.-K. (2006). Regulation of antibacterial defense in the small intestine by the nuclear bile acid receptor. *Proceedings of the National Academy of Sciences of the United States of America*.

[26] Bernstein H., Bernstein C., Payne C. M., Dvorakova K., Garewal H. (2005). Bile acids as carcinogens in human gastrointestinal cancers. *Mutation Research—Reviews in Mutation Research*.

[27] Baptissart M., Vega A., Maqdasy S. (2013). Bile acids: from digestion to cancers. *Biochimie*.

[28] Yokota A., Fukiya S., Ooka T., Ogura Y., Hayashi T., Ishizuka S. (2012). Is bile acid a determinant of the gut microbiota on a high-fat diet?. *Gut Microbes*.

[29] Stenman L. K., Holma R., Eggert A., Korpela R. (2013). A novel mechanism for gut barrier dysfunction by dietary fat: epithelial disruption by hydrophobic bile acids. *The American Journal of Physiology—Gastrointestinal and Liver Physiology*.

[30] Devkota S., Wang Y., Musch M. W. (2012). Dietary-fat-induced taurocholic acid promotes pathobiont expansion and colitis in *II*10^−/−^ mice. *Nature*.

[31] Schoenfield L. J., Lachin J. M., Baum R. A. (1981). Chenodiol (chenodeoxycholic acid) for dissolution of gallstones: the national cooperative gallstone study. A controlled trial of efficacy and safety. *Annals of Internal Medicine*.

[32] Watanabe M., Houten S. M., Wang L. (2004). Bile acids lower triglyceride levels via a pathway involving FXR, SHP, and SREBP-1c. *The Journal of Clinical Investigation*.

[33] Yamagata K., Daitoku H., Shimamoto Y. (2004). Bile acids regulate gluconeogenic gene expression via small heterodimer partner-mediated repression of hepatocyte nuclear factor 4 and Foxo1. *Journal of Biological Chemistry*.

[34] Horton J. D., Goldstein J. L., Brown M. S. (2002). SREBPs: activators of the complete program of cholesterol and fatty acid synthesis in the liver. *Journal of Clinical Investigation*.

[35] King T. S., Elia M., Hunter J. O. (1998). Abnormal colonic fermentation in irritable bowel syndrome. *The Lancet*.

[36] Anderson M. L., Pasha T. M., Leighton J. A. (2000). Eradication of small intestinal bacterial overgrowth reduces symptoms of irritable bowel syndrome. *The American Journal of Gastroenterology*.

[37] DuPont A. W. (2008). Postinfectious irritable bowel syndrome. *Clinical Infectious Diseases*.

[38] Gwee K.-A., Leong Y.-L., Graham C. (1999). The role of psychological and biological factors in postinfective gut dysfunction. *Gut*.

[39] Liebregts T., Adam B., Bredack C. (2007). Immune activation in patients with irritable bowel syndrome. *Gastroenterology*.

[40] Langhorst J., Junge A., Rueffer A. (2009). Elevated human beta-defensin-2 levels indicate an activation of the innate immune system in patients with irritable bowel syndrome. *The American Journal of Gastroenterology*.

[41] Brint E. K., MacSharry J., Fanning A., Shanahan F., Quigley E. M. M. (2011). Differential expression of toll-like receptors in patients with Irritable bowel syndrome. *The American Journal of Gastroenterology*.

[42] Nobaek S., Johansson M.-L., Molin G., Ahrné S., Jeppsson B. (2000). Alteration of intestinal microflora is associated with reduction in abdominal bloating and pain in patients with irritable bowel syndrome. *The American Journal of Gastroenterology*.

[43] Niedzielin K., Kordecki H., Birkenfeld B. (2001). A controlled, double-blind, randomized study on the efficacy of Lactobacillus plantarum 299V in patients with irritable bowel syndrome. *European Journal of Gastroenterology and Hepatology*.

[44] Kim H. J., Camilleri M., McKinzie S. (2003). A randomized controlled trial of a probiotic, VSL#3, on gut transit and symptoms in diarrhoea-predominant irritable bowel syndrome. *Alimentary Pharmacology and Therapeutics*.

[45] Malinen E., Krogius-Kurikka L., Lyra A. (2010). Association of symptoms with gastrointestinal microbiota in irritable bowel syndrome. *World Journal of Gastroenterology*.

[46] Balsari A., Ceccarelli A., Dubini F., Fesce E., Poli G. (1982). The fecal microbial population in the irritable bowel syndrome. *Microbiologica*.

[47] Si J. M., Yu Y. C., Fan Y. J., Chen S. J. (2004). Intestinal microecology and quality of life in irritable bowel syndrome patients. *World Journal of Gastroenterology*.

[48] Mättö J., Maunuksela L., Kajander K., Palva A., Saarela M. (2005). Composition and temporal stability of gastrointestinal microbiota in irritable bowel syndrome—a longitudinal study in IBS and control subjects. *FEMS Immunology and Medical Microbiology*.

[49] Swidsinski A., Weber J., Loening-Baucke V., Hale L. P., Lochs H. (2005). Spatial organization and composition of the mucosal flora in patients with inflammatory bowel disease. *Journal of Clinical Microbiology*.

[50] Duboc H., Rainteau D., Rajca S. (2012). Increase in fecal primary bile acids and dysbiosis in patients with diarrhea-predominant irritable bowel syndrome. *Neurogastroenterology and Motility*.

[51] Ridlon J. M., Kang D.-J., Hylemon P. B. (2006). Bile salt biotransformations by human intestinal bacteria. *Journal of Lipid Research*.

[52] Odunsi-Shiyanbade S. T., Camilleri M., McKinzie S. (2010). Effects of chenodeoxycholate and a bile acid sequestrant, colesevelam, on intestinal transit and bowel function. *Clinical Gastroenterology and Hepatology*.

[53] Arasaradnam R. P., Covington J. A., Harmston C., Nwokolo C. U. (2014). Review article: Next generation diagnostic modalities in gastroenterology—gas phase volatile compound biomarker detection. *Alimentary Pharmacology and Therapeutics*.

[54] Bäckhed F., Ley R. E., Sonnenburg J. L., Peterson D. A., Gordon J. I. (2005). Host-bacterial mutualism in the human intestine. *Science*.

[55] Arasaradnam R. P., Ouaret N., Thomas M. G. (2013). A novel tool for noninvasive diagnosis and tracking of patients with inflammatory bowel disease. *Inflammatory Bowel Diseases*.

[56] Covington J. A., Westenbrink E. W., Ouaret N. (2013). Application of a novel tool for diagnosing bile acid diarrhoea. *Sensors*.

